# Association of Blood Pressure with Metabolic Factors, Stress Levels, Physical Activity, and Nutrient Intake in Overweight or Obese Ecuadorian University Students: A Study Based on Mediation Analysis

**DOI:** 10.3390/nu18020201

**Published:** 2026-01-08

**Authors:** María Alejandra Aguirre-Quezada, María Pilar Aranda-Ramírez, María del Carmen-García, Geovanny Reiván-Ortiz

**Affiliations:** 1 Nursing Career, Catholic University of Cuenca, Azogues 18071, Ecuador; maaguirreq@ucacue.edu.ec; 2 Department of Physiology, University of Granada, 18071 Granada, Spain; mcgrios@ugr.es; 3 Psychologic Career, Catholic University of Cuenca, Azogues 18071, Ecuador; greivano@ucacue.edu.ec

**Keywords:** nutrients, metabolic health, stress, blood pressure, mediation analysis

## Abstract

Background: Obesity is a worldwide public health problem, affecting organs and systems. It is also a cardiovascular risk factor, which facilitates the development of diseases, such as arterial hypertension, dyslipidemia, and diabetes, which are used as criteria for the diagnosis of metabolically unhealthy obesity. Objective: To analyze the association between blood pressure and metabolic health factors, stress level, and nutrient intake in overweight and obese university students through mediation analysis. Methods: A quantitative, non-experimental, cross-sectional, correlational, and quantitative study was conducted in a sample of 230 obese/overweight university students selected by a multistage mass random sampling method. To evaluate habitual dietary intakes, a CFCA food frequency questionnaire was applied; a DASS-21 scale was used to evaluate stress; blood pressure and anthropometric data were collected; insulin levels, lipid profile, and glucose were determined using fasting blood samples. Statistical analysis was performed using univariate methods (frequencies, trend, and dispersion measures) and a mediational model. Results: The majority were young people aged 18 years (18.7%), with morning and afternoon shifts (60%), overweight (76.1%), and obese (23.9%). Not all obese people have arterial hypertension; however, an increase in BMI increases the risk of suffering from this disease. Model 1 showed that certain types of stress and sex at birth have an important relationship with diastolic blood pressure, mediated in some cases by weight. In Model 2, weight is a significant mediator in the relationship between moderate stress and systolic BP, and between sex at birth and systolic BP, thus allowing us to contribute to the understanding of how these variables are interrelated. Conclusions: This suggests that severe stress and sex at birth not only affect BP directly, but also do so through their effect on weight. Thus, both pathways contribute to understanding the relationship between stress, sex at birth, and diastolic and systolic blood pressure. Nevertheless, the results of this study provide empirical knowledge to design evidence-based prevention and treatment strategies.

## 1. Introduction

Over the past four decades, obesity has become one of the leading threats to public health worldwide. Over the last 45 years, its prevalence has tripled, currently affecting 39% of adults with overweight and 13% with obesity [1]. The World Obesity Federation warns that, unless effective prevention policies are implemented, more than half of the global population will experience overnutrition in the coming years [2]. This phenomenon not only increases the burden of disease, but also places growing pressure on already overstretched healthcare systems, with projections estimating that obesity related costs will exceed 3% of global gross domestic product in 161 countries by 2060 [3].

At the same time, the world is undergoing an epidemiological transition characterized by the predominance of cardiovascular diseases as the leading cause of global mortality, surpassing infectious diseases [4]. This shift reflects profound changes in contemporary lifestyles, marked by unhealthy dietary patterns, physical inactivity, and a growing burden of mental health conditions, particularly psychosocial stress especially among young populations [5]. In this context, obesity, now categorized as a chronic disease, is directly associated with the development of multiple cardiometabolic disorders.

Excess body weight thus constitutes an integrative marker of cardiometabolic risk, as it modulates metabolic alterations, such as insulin resistance, dyslipidemia, and hyperglycemia, in specific profiles [6]. Unlike biochemical indicators such as HOMA-IR or fasting glucose, which may not be fully expressed in young adults, this anthropometric measure reflects the cumulative impact of behavioral, psychological, and physiological exposures over time and maintains a direct and consistent relationship with blood pressure. In this sense, obesity not only predicts future risk but may also act as a central node through which multiple determinants of cardiovascular health interact [7,8].

Contemporary scientific evidence strongly supports the classification of obesity as a chronic disease rather than merely a risk factor [9]. Epidemiological studies have shown that hypertension is up to six times more prevalent in individuals with obesity compared with those with a normal body mass index [10], and between 60% and 70% of hypertension cases in adults are directly attributable to adiposity [11]. Global projections estimate that the prevalence of hypertension will reach 29% by 2025, affecting more than 1.5 billion people, despite being a preventable condition [12]. The sustained rise in obesity has been identified as one of the main drivers of this trend [13].

From Bluher’s pathophysiological perspective, the relationship between obesity and hypertension is highly complex. Adipose tissue is metabolically active and plays a key role in regulating inflammatory, hormonal, and hemodynamic processes [14,15]. Recent research suggests that it is not merely the amount of body fat but rather adipose tissue dysfunction that may explain the mechanistic link between obesity and adverse health outcomes [14]. Genetic, environmental, and behavioral factors interact with this dysfunction, causing rise to heterogeneous trajectories of cardiometabolic risk. Within this framework, important questions remain regarding the specific mechanisms through which weight gain induces cardiovascular alterations and the extent to which these conditions may be reversible through early interventions [15].

Several studies have documented that even modest weight gain during early adulthood has a significant impact on blood pressure. Gong et al. demonstrated that a 5 kg increase in body weight is associated with a 2.8-fold higher risk of developing hypertension [16]. Similarly, longitudinal studies among Japanese university students have shown that changes in body weight exert a direct and rapid effect on blood pressure, even over relatively short periods [17]. These findings highlight the vulnerability of young adults and the importance of intervening before pathological trajectories become established.

Beyond body weight, behavioral and psychosocial factors play a crucial role in blood pressure regulation. High sodium intake, characteristic of industrialized diets with low nutritional value, contributes to extracellular volume expansion and represents a well-established risk factor for hypertension and cardiovascular disease [18]. The World Health Organization recommends limiting sodium intake to less than 2 g per day, a target that has been shown to reduce all-cause mortality when sustained over time [19]. Similarly, chronic stress, physical inactivity, alcohol and tobacco use, and inadequate sleep patterns have been consistently associated with elevated blood pressure, particularly in university settings [20,21].

Among university student populations, this issue is particularly relevant. Globally, the prevalence of overweight in this group ranges from 20% to 40% [22,23]. The transition to university life is often accompanied by substantial changes in dietary and physical activity habits, characterized by monotonous, hypercaloric, and high-sodium diets, low intake of fiber and micronutrients, sedentary behavior, and high exposure to academic stress [24,25]. Recent studies conducted in diverse cultural contexts have reported a high prevalence of undiagnosed prehypertension and hypertension among university students, which is associated with perceived stress, sleep deprivation, and risky behaviors [26].

Despite the growing body of international evidence, most studies have examined the relationship between obesity, blood pressure, and metabolic factors in isolation, without simultaneously integrating key dimensions such as stress, nutrient intake, and physical activity [27,28]. Moreover, much of the available evidence originates from high-income countries or distinct sociocultural contexts, limiting its applicability to emerging economies [29]. In Ecuador, a significant gap in local evidence persists, hindering the accurate characterization of the epidemiological profile of university students while accounting for their cultural, dietary, and lifestyle characteristics.

Against this backdrop, generating local data is essential to understand not only the direct associations between obesity and blood pressure but also the indirect mechanisms through which metabolic, nutritional, behavioral, and psychosocial factors interact. Mediation analysis models offer a robust analytical approach to explore these complex pathways, identify key intervention targets, and provide an empirical basis for designing preventive strategies tailored to the Ecuadorian context.

Within this framework, the present study aimed to analyze the association between blood pressure, metabolic health factors, stress levels, nutrient intake, and physical activity among overweight and obese university students in Ecuador, using a mediation analysis approach. Given that overweight and obesity are major contributors to global mortality and disability [30,31], identifying their direct and indirect predictors in young populations is essential for informing public policies, strengthening primary prevention, and optimizing clinical management protocols at the primary healthcare level.

## 2. Materials and Methods

### 2.1. Design and Participants

Epidemiological, nonexperimental, cross-sectional, correlational, quantitative, developed in the city of Azogues, Ecuador. The estimated sample size was calculated based on a 60% prevalence of obesity among overweight and obese Ecuadorian students. The unit of analysis was the overweight and obese students of the Catholic University of Cuenca, which corresponds to 230 students of different socioeconomic levels, power of 80%, desired confidence interval of 0.95, type I error of 0.05, and precision (d) of 7%. The period covered was March 2023 to March 2025. According to the university age range, participants between 18 and 28 years were chosen through a multistage mass sampling approach.

### 2.2. Inclusion and Exclusion Criteria

All students who, voluntarily agreed to sign the informed consent form were, included, excluding students with pregnancy, endocrine or genetic disorders (hypothyroidism, type 1 diabetes mellitus, and Cushing’s syndrome), following a weight-loss diet, taking mineral and vitamin supplements, or medications that could affect blood glucose, lipid profile, body weight, or blood pressure.

### 2.3. Data Acquisition

Information was collected using observation and survey techniques.

#### 2.3.1. Anthropometry

The dependent variable was the nutritional status of the students, obtained through the body mass index (BMI), which is an indicator of the relationship between weight in kg and height in m^2^, used to classify overweight and obesity, according to the WHO: Normal weight = BMI 25 kg/m^2^. Overweight = BMI equal to or greater than 25 kg/m^2^. Obesity = BMI equal to or greater than 30 kg/m^2^.

#### 2.3.2. Socioeconomic Variables

The independent variables were categorized as socioeconomic. The Gaffar scale was applied to evaluate the socioeconomic status. For, the sociodemographic variables, age, sex at birth, marital status, academic unit, career, and study schedule of the participants were collected through a questionnaire.

#### 2.3.3. Assessment of Dietary Intake

The dietary intake of the participants was measured using a food frequency questionnaire (CFCA) [32]. A trained nutritionist completed the questionnaires and asked people to indicate the frequency of food consumption (daily, weekly, or monthly) and the amount consumed (based on common standard portion sizes) in the last 12 months. Then, using home measurements, all reported values were converted into grams per day. Subsequently, the total energy and nutrient intake of each individual was calculated by summing the energy and nutrients from all foods. To derive nutrient intake, grams of food consumption were entered into Nutrimind software 2024 [33].

#### 2.3.4. Cardiometabolic Risk Factors

The evaluation of anthropometric indicators and cardiometabolic risk factors was carried out by a previously trained team from the nursing faculty, who recorded the anthropometric indices of all participants. Weight was measured using a calibrated electronic scale (BCS-G6) (closest to 0.1 kg). Standing height was recorded using a stadiometer (nearest 0.1 cm). Waist circumference (WC) was measured twice for each participant, and the mean value of two assessments was considered WC [34]. After a 5 min rest, diastolic blood pressure (DBP) and systolic blood pressure (SBP) were recorded twice with a 15 min recovery interval, on the right arm [35].

The average of two measurements was considered in the analysis. To determine the biochemical values, venous blood samples were obtained in a seated position, after a 12 h. Fasting blood glucose concentration, lipid profile, and insulin concentrations were determined. To estimate insulin resistance, Homeostasis Model Assessment Insulin Resistance (HOMA-IR) was calculated using the following formula: HOMA-IR = [insulin (µIU/mL) × glucose (mg/dL)]/405 [36].

#### 2.3.5. Stress Level

For the evaluation of stress level, the abbreviated version of the Depression Anxiety and Stress Scales (DASS-21) was used [37]. Each item was answered according to the presence and intensity of each symptom on a Likert-type response scale from 0 to 3 points. Each scale has seven items, and its total score is calculated with the sum of the items belonging to that scale and ranges from 0 to 21 points.

#### 2.3.6. Level of Physical Activity

Finally, the level of physical activity of each participant was assessed using the Physical Activity Questionnaire (IPAQ) [38], which included nine questions on various activities during weekdays and weekends. Based on total scores, undergraduates were classified as active (score ≥ 3), not very active (3 < score ≤ 2), or sedentary (or no regular weekly activity) (score < 2).

### 2.4. Data Analysis

For the analysis of the first objective, a descriptive analysis was conducted. Qualitative variables were presented as frequencies (absolute and relative), whereas quantitative variables were analyzed using measures of central tendency (mean and median) and dispersion (standard deviation and interquartile range). The Mann–Whitney U test and the chi-square test was used to analyze group differences, and baseline characteristics were compared according to hypertension status to identify possible differences between hypertensive and non-hypertensive participants and to explore variables that could act as confounders or modifiers in the association between obesity and blood pressure.

For the second objective, which aimed to evaluate the relationships between stress, body weight, and blood pressure (systolic and diastolic), a mediation analysis was performed to explore the mediating effect of body weight on the associations between independent and dependent variables.

In the model design, the path included stress (measured as mild, moderate, and severe stress) and sex at birth as independent variables, body weight as the mediating variable, and systolic and diastolic blood pressure as dependent variables. Causal relationships were proposed, considering that stress and sex at birth could influence blood pressure directly and through their impact on body weight.

Model coefficients were estimated using the maximum likelihood technique with the statistical software Jamovi v2.2.2 [39]. Standard errors and *p*-values were calculated to assess the statistical significance of the effects. 95% confidence intervals for indirect effects were obtained using the standard Delta method, providing robust estimates of mediated relationships. A *p*-value < 0.05 was considered statistically significant. Additionally, fully standardized coefficients (β) facilitate the interpretation of effect sizes. Finally, to verify model fit, indices such as the chi-square test, Comparative Fit Index (CFI), and Root Mean Square Error of Approximation (RMSEA) were used. A CFI > 0.90 and RMSEA < 0.08 were considered indicators of good model fit.

### 2.5. Ethical Responsibility

This study adheres to the principles of the Declaration of Helsinki and is part of the research project titled *“Intake Patterns, Psychological Patterns, and Metabolic Health”*. The project was approved by the Bioethics Committee for Human Research at the Catholic University of Cuenca (CEISH—UCACUE) under the code CEISH—UCACUE—045. The authors declare no conflicts of interest.

## 3. Results

When analyzing the data presented in Table 1, it can be identified that most of the subjects were18 years old, male, single, half of the participants were from the Academic Unit of Health and Wellness, from the morning and afternoon study days, and from a high social stratum. In addition to identifying a low level of physical activity in more than 80% of the participants.

When comparing the study variables across blood pressure classification groups, significant differences were identified, as shown in Table 2. Students with grade 1 hypertension exhibit a more impaired metabolic profile than those with optimal blood pressure. They have higher body weight, waist circumference, glucose, insulin, and insulin resistance, as well as a more adverse lipid profile characterized by elevated total cholesterol and triglycerides, along with lower HDL levels.

The results regarding nutrient intake in the groups are summarized in Table 3. Although no statistically significant differences were found, some observations are clinically relevant. Caloric intake, as well as consumption of protein, carbohydrates, lipids including saturated, monounsaturated, and polyunsaturated fatty acids, fiber, and water, were similar in the two groups.

One notable finding is that cholesterol intake exceeded the recommended 300 mg/day, contributing to an unfavorable lipid profile. Additionally, the average fiber intake was below the daily recommendation, particularly in the group with higher blood pressure.

Vitamin intake is detailed in Table 4. Although no statistically significant differences were found for vitamins A, thiamine, riboflavin, pyridoxine, cobalamin, pantothenic acid, biotin, C, D, E, and folic acid, this suggests that students in both groups have similar intake levels, and these micronutrients do not appear to influence the observed differences in blood pressure within the study population.

In contrast, niacin intake showed a significant difference, with students in the grade 1 hypertensive group consuming higher amounts of this vitamin. The higher niacin intake may be linked to the consumption of niacin rich foods, such as meats and fortified products.

Table 5 describes the mineral intake. Although no statistically significant differences were found, the observed intake patterns and their potential impact on metabolic and cardiovascular health in this high-risk population are noteworthy.

The HAG1 group consumes more sodium than the optimal blood pressure group. Given that excess sodium intake is strongly associated with hypertension, this trend suggests that higher sodium consumption may contribute to increased blood pressure in these students.

For potassium, calcium, phosphorus, magnesium, iron, zinc, iodine, copper, selenium, and chlorine, intake levels were similar between groups, indicating that these minerals do not explain the observed differences in blood pressure.

Manganese intake was slightly higher in the hypertensive group. Although this difference did not reach statistical significance, it suggests a possible relationship between manganese intake and hypertension, which warrants further investigation for confirmation.

An analysis of physical activity levels revealed a common pattern across both groups, indicating that physical activity is not a distinguishing factor in the presence of hypertension in this population. However, low physical activity remains an important risk factor for overall cardiovascular health.

In contrast, elevated stress levels were clearly associated with the presence of grade 1 hypertension in this population.

These finding highlights that this group exhibits both low physical activity and high stress levels, which together significantly contribute to hypertension and obesity, as described in Table 6.

**Figure 1:**
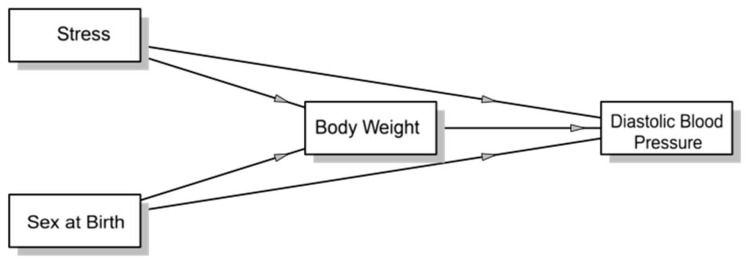
Mediation analysis for diastolic blood pressure in university students.

Figure 1 and Table 7 present, the mediation analysis of diastolic blood pressure, highlighting the presence of both direct and indirect effects of the analyzed variables. Moderate stress exerts a significant and positive indirect effect on diastolic blood pressure through body weight, indicating that moderate stress levels may contribute to increased blood pressure by promoting weight gain. In contrast, severe stress has a significant and positive direct effect on diastolic blood pressure, suggesting a direct association between severe stress and increased blood pressure.

Mild stress does not show significant direct or indirect effects on diastolic blood pressure, although its indirect effect through weight is marginally significant. Notably, sex at birth has a significant negative indirect effect on diastolic blood pressure, indicating that this variable may influence blood pressure primarily through its impact on body weight.

Overall, the findings suggest that certain types of stress and sex at birth are significantly related to diastolic blood pressure, with weight acting as a mediator in some cases. However, the effects vary depending on the type of stress, with severe stress having the most pronounced and direct impact on the dependent variable.

Indirect effects illustrate how weight mediates the relationship between independent variables and diastolic blood pressure. There is no evidence that weight acts as a significant mediator between mild stress and diastolic blood pressure. However, a significant mediating effect of weight was observed between moderate stress and diastolic blood pressure, confirming that stress primarily influences blood pressure through its impact on body weight.

Weight has a direct and significant impact on diastolic blood pressure, supporting existing evidence that increased body weight, particularly visceral fat accumulation, contributes to elevated diastolic blood pressure through the activation of the renin–angiotensin–aldosterone system and increased vascular resistance.

No significant direct effect of sex at birth on diastolic blood pressure was observed, indicating that its influence occurs primarily through weight. Interventions should focus on weight reduction to mitigate the effects of stress and sex-related differences in blood pressure.

**Figure 2:**
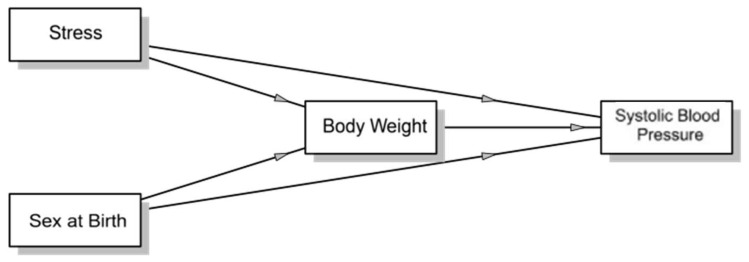
Mediation analysis for systolic blood pressure in university students.

Figure 2 and Table 8 show that there is no evidence supporting weight as a significant mediator between mild stress and systolic blood pressure. However, a significant mediating effect of weight was found between moderate stress and systolic blood pressure, suggesting that this type of stress may influence the dependent variable through its impact on body weight. In contrast, no significant mediating effect of weight was observed between severe stress and systolic blood pressure, indicating that severe stress does not exert a relevant indirect impact on systolic blood pressure through weight. Additionally, a significant negative mediating effect of weight was found in the relationship between sex at birth and systolic blood pressure.

In the mediation model, weight has a direct and significant impact on systolic blood pressure, supporting evidence that increased weight contributes to higher systolic blood pressure through the activation of the renin–angiotensin–aldosterone system and increased vascular resistance.

No significant relationship was identified between mild or severe stress and weight. However, when analyzing moderate stress, a significant and positive association with weight was observed, indicating that this type of stress may trigger weight gain mechanisms. Additionally, a strong negative relationship was found between sex at birth and weight, reflecting inherent metabolic differences between sexes, such as body composition, hormone levels, and basal metabolic rates.

The direct effects provide important insights into the realities of university students in Ecuador. The analysis determined that moderate stress and sex at birth have no direct effect on systolic blood pressure, as their impact is primarily mediated by weight. This contrasts with the finding that severe stress has a significant and positive direct effect on systolic blood pressure.

Ultimately, the study confirms that weight is a significant mediator in the relationships between moderate stress and systolic blood pressure, as well as sex at birth and systolic blood pressure.

This study found that severe stress has a significant impact on blood pressure, mediated by weight. Weight emerged as the most relevant mediator in the relationship between stress, sex at birth, and blood pressure, suggesting that students experiencing high stress levels may gain weight, which in turn could lead to increased blood pressure.

## 4. Discussion

The significance of this study lies in identifying factors that can influence student behaviors in a way that reduces the risks associated with high blood pressure. The expected relationships were identified between waist circumference, cholesterol, triglycerides, HDL, insulin, stress levels, and glucose, consistent with previous studies. These findings provide evidence to clarify the relationship between these variables among Ecuadorian students and align with the results reported by Heindel et al., who demonstrated that the university environment increases the risk of weight gain [40], a similarity reflected in Table 2.

In line with this, a study conducted in India found that most participants were male, with an average age of 18.57 years, and 19% were overweight. Based on sex-specific and waist circumference cutoff points, approximately 5% and 21% of participants were at a substantially higher risk of metabolic complications [40]. These findings are also consistent with those of Luis Rangel et al., who examined the association between overweight, obesity, cholesterol, blood pressure, and diabetes in Panamanian university students, determining that 46.12% of the study population was classified as overweight or obese [41]. These results align with the findings in Table 1 and Table 2.

Similarly, Hemmingsson et al. [42] in Sweden noted that most studies indicate an inverse relationship between physical activity (PA) and body mass index (BMI). However, the impact of obesity on this relationship remains unclear. Their cross-sectional study demonstrated that BMI was significantly associated with physical activity in obese individuals, which, when compared to the present study, may suggest a contrast in the identification of this variable.

In contrast, Yarizadeth et al. identified nutrient intake patterns that differed from the present study’s findings, particularly higher intake levels of fiber, protein, and saturated fats [43]. These discrepancies likely indicate dietary differences between European, Asian, and American populations, which may be influenced by variations in development levels and socioeconomic factors [44].

The study’s findings show similarity to publications from Chilean university students. It must be acknowledged that there is evidence documenting nutritional imbalances in university students and an urgent need for educational and nutritional interventions [45]. However, the nutritional profile of university students and the impact of their diet on this profile remain largely unknown. This research determined low fiber consumption, with only 16% reaching the recommended intake, generating nutritional risk due to deficiency in the study population.

Regarding sociodemographic, anthropometric, cardiometabolic, and stress-related differences in blood pressure, this study has generated several important insights. A comparison with the study by Koncar et al. [46] revealed a predominantly non-dipping blood pressure pattern in obese individuals, regardless of obesity severity, a finding consistent with the present study. In a Chinese population, it was also concluded that triglyceride and glucose levels are independently associated with the incidence of hypertension [47]. The work carried out by Sabadabi et al. concluded that a lower socioeconomic status was associated with metabolic syndrome and hypertension, while obesity was associated with a higher socioeconomic status. This shows a certain similarity with the results of this research, indicating that it may be necessary to develop personalized preventive policies for populations of each socioeconomic class [48].

The positive relationship between blood pressure and stress observed in this study is consistent with findings from other studies [49,50], which indicate that stress increases appetite and food intake, particularly carbohydrates, due to elevated ghrelin and cortisol levels, leading to greater hunger and a preference for energy-dense foods [51]. Another notable finding was the negative relationship between fat and sodium intake and stress, a connection supported by research suggesting that stress can significantly affect eating habits and nutrient processing. For instance, while some individuals under stress seek comfort foods, others reduce their fat and sodium intake as a way to manage their health or due to changes in appetite [52,53].

A study conducted in Japan on university students showed that changes in body weight have a significant impact on blood pressure and highlights the potential public health benefits of strategies aimed at preventing weight gain throughout adulthood. This study examined the longitudinal impact of weight change on blood pressure. Weight gain increased ΔSBP and ΔDBP, while weight loss decreased them [17]. The results confirm the relationship between weight and blood pressure [54]. Individuals with obesity often exhibit a metabolic profile characterized by insulin resistance, inflammation, and dyslipidemia, which can influence nutrient metabolism differently compared to individuals with hypertension alone [55,56].

Additionally, several studies support the usefulness of mediation analysis in investigating factors such as eating behavior, domestic violence, and dietary phytochemicals [55,56,57]. This presents an innovative methodology for studying these phenomena, enabling the identification of latent structures in variables related to blood pressure, overweight, and obesity through these models.

The findings propose a mediation analysis framework for blood pressure, integrating variables that have been previously studied in isolation [58,59]. For example, predictive models incorporating variables similar to those in this study have included factors such as body fat distribution (visceral fat and waist-to-hip ratio) [60] and insulin resistance [61].

Previous research by Sasha Yu et al. [62] demonstrated the causal and independent impact of education on hypertension and blood pressure, identifying cardiometabolic mediators as key targets for hypertension prevention among individuals with lower education levels. These authors emphasized the importance of education level as a key explanatory mechanism for understanding behavioral modifications affecting blood pressure.

## 5. Limitations

Regarding the limitations of the study, it is necessary to mention that the nutritional assessment by anthropometry based on weight, height, and BMI may not reflect the proportion of body fat and its distribution and could represent a limitation on the findings of the differences in risk between men and women, which may lead to inaccurate conclusions regarding the associations with cardiometabolic characteristics.

On the other hand, the use of an intake frequency questionnaire may, induce recall biases, especially in a young population that may not remember or adequately report their dietary habits. Furthermore, the accuracy of portions and daily variations depends on self-reporting, which affects the accurate estimation of caloric intake and macronutrient ratio. This could affect the accuracy in identifying reported nutrient patterns.

Tests for glucose, insulin, triglycerides, cholesterol, and HDL are, performed at a specific point in time and may not reflect variability over time. This may limit the accuracy in estimating HOMA-IR and in assessing participants’ metabolic risk. In addition, because of the significant role of vitamin and mineral status in disease prevention, it is important to identify deficiencies in populations with obesity through plasma determination. Information that was not considered in our study.

By creating a predictive model that includes multiple variables, there is a risk of overfitting the model to the specific study sample, which may limit its applicability to other university populations or age groups.

The study was cross-sectional, so risk factors and blood pressure were assessed simultaneously, which could introduce bias. To establish a causal relationship in the future, a longitudinal study should be conducted.

It should be noted that the baseline generated for defining the level of anxiety in university students corresponds to a post-pandemic contribution, considering that this condition was very complex for the target population. However, no pattern of comparison with pre-pandemic studies in Ecuador has been defined.

These limitations could be assessed in future research to make the results more consistent, even with longitudinal designs. In future work, it would be important to consistently detail the comorbidities and previously diagnosed mental health illnesses of the participants, as the study posed a cross-sectional approach.

## 6. Strengths

The study stands out for its contribution with the integration of metabolic variables, level of stress, and intake, specific to Ecuador, which has also allowed for the characterization of a phenomenon from a multicausal viewpoint. For the first time, a nutrient-based consumption profile is available for the overweight and obese population, which, although it shows a difference with other studies, has generated a reference that describes the cultural behavior in the study population.

The use of consumption frequency questionnaires is undeniably an effective tool for large-scale observational studies, accessible, time-efficient, and provides an overview of the dietary habits of the population without requiring continuous follow-up.

The evaluation of laboratory tests, on the other hand, provides a solid basis for assessing the metabolic profile of the participants.

The inclusion of stress allows a multidimensional analysis of the health status university students. Based on the influence on cardiometabolic risk, the study incorporates an integral and innovative perspective and greater relevance and sensitivity to the characteristics of the sample, improving the quality and applicability of the results.

## 7. Conclusions

This study integrates nutritional, metabolic, stress, and physical activity aspects, providing a comprehensive and detailed overview of the health status of the study population.

When comparing the groups, it was observed that participants with hypertension had higher body weight, larger waist circumference, higher glucose and insulin levels, greater insulin resistance (HOMA-IR), and a more unfavorable lipid profile—high total cholesterol and triglyceride levels and low HDL levels. These results suggest that obesity, especially visceral obesity, and insulin resistance play a central role in the genesis of hypertension in this cohort.

It was identified that increased body weight raises blood pressure, and the adjustment of the proposed model allows for the mediation of weight on systolic and diastolic blood pressure to be explained. This study provides additional evidence on the complex relationship between stress, sex at birth, and blood pressure. The results also suggest that severe stress affects blood pressure, contributing evidence to the country’s epidemiological profile through a variable that has been little studied in public theories in combination.

Although the underlying mechanisms have been described previously, the specific contribution to the Ecuadorian context focuses on the provision of original data that could support the planning of localized prevention strategies in the medium term.

Disseminating these results may contribute to reducing the future incidence of cardiovascular disease. In addition, documenting the presence of risk factors in this age group provides essential local evidence for the design of prevention programs.

## Figures and Tables

**Table 1:** Characterization of the study population.

Dimension	Variable	*f*	%
Sex at birth	Male	133	58
	Female	97	42
Marital status	Single	188	82
	Married	26	11
	Unmarried	10	4.4
	Divorced	5	2.2
	Widowed	1	0.4
Academic Unit	Health and Wellness	115	50
	Education and Humanities	41	18
	Social Sciences	36	16
	Engineering and construction	23	10
	Administrative and business sciences	15	6.6
Study schedule	Mornings and afternoons	138	60
	Evening	50	22
	Morning	42	18
Social stratum	Stratum I	84	36
	Stratum II	65	28
	Stratum III	70	31
	Stratum IV	11	4.8
Level of physical activity	Low physical activity	200	87%
	Moderate physical activity	30	13%

**Table 2:** Relationship of blood pressure to metabolic health.

Characteristic	NBP	HAG1	*p*-Value
	1 N = 172 (75%)	2 N = 58 (25%)	
Weight (kg)	73 (65, 79)	79 (72, 90)	<0.001
Waist circumference (cm)	88 (84, 95)	95 (89, 102)	<0.001
Glucose (mg/dL)	94 (91, 97)	99 (96, 118)	<0.001
Insulin (U/mL)	10.33 (9.20, 11.00)	10.88 (10.40, 11.88)	<0.001
Cholesterol (mg/dL)	180 (154, 210)	223 (197, 254)	<0.001
Triglycerides (mg/dL)	154 (103, 163)	169 (157, 183)	<0.001
HDL (mg/dL)	44.78 (43.04, 46.86)	42.94 (40.43, 45.22)	<0.001
HOMA	2.37 (2.15, 2.69)	3.05 (2.48, 3.42)	<0.001

**Table 3:** Relationship of nutrient intake to blood pressure groups.

Characteristic	NBP	HAG1	*p*-Value
	1 N = 172 (75%)	2 N = 58 (25%)	
Kcal	2256 (2130, 2367)	2219 (2100, 2327)	0.42
Cholesterol (mg)	356 (308, 428)	356 (304, 429)	0.88
Fiber (g)	21 (18, 27)	20 (18, 25)	0.24
Proteins (g)	71 (58, 84)	76 (66, 86)	0.20
Carbohydrates (g)	298 (269, 331)	294 (260, 325)	0.34
Lipids (g)	88 (82, 94)	85 (78, 96)	0.37
Monounsaturated fatty acids (g)	41 (36, 46)	41 (37, 44)	0.56
Polyunsaturated fatty acids (g)	8.82 (7.51, 10.07)	8.53 (7.16, 9.89)	0.60
Saturated fatty acids (g)	30 (24, 38)	29 (21, 36)	0.10
Water (mL)	1478 (1258, 1687)	1569 (1287, 1705)	0.55

**Table 4:** Relationship of vitamin intake to blood pressure groups.

Characteristic	NBP	HAG1	*p*-Value
	1 N = 172 (75%)	2 N = 58 (25%)	
Vitamin A (µg)	603 (470, 890)	682 (562, 809)	0.15
Thiamine (vitamin B1) (mg)	1.18 (1.00, 1.66)	1.25 (1.02, 1.60)	0.60
Riboflavin (vitamin B2) (mg)	1.86 (1.67, 2.05)	1.89 (1.65, 2.18)	0.67
Pyridoxine (vitamin B6) (mg)	1.58 (1.40, 1.97)	1.67 (1.41, 2.10)	0.72
Cobalamin (vitamin B12) (µg)	13 (3, 20)	13 (4, 19)	0.90
Vitamin C (mg)	159 (92, 210)	115 (86, 246)	0.55
Vitamin D (µg)	2.17 (1.04, 2.58)	1.98 (1.51, 5.41)	0.39
Vitamin E (mg)	9.8 (7.6, 12.8)	9.6 (8.1, 11.9)	0.49
Niacin (vitamin B3) (mg)	17.5 (13.4, 22.8)	20.7 (16.2, 26.4)	0.027
Pantothenic acid (vitamin B5) (mg)	3.90 (3.23, 4.98)	4.10 (3.16, 5.04)	0.93
Biotin (mg)	3.54 (2.71, 4.78)	3.67 (2.68, 4.85)	0.86
Folic acid (vitamin B9) (µg)	304 (234, 348)	299 (229, 357)	0.94

**Table 5:** Relationship of mineral intake to blood pressure groups.

Characteristic	NBP	HAG1	*p*-Value
	1 N = 172 (75%)	2 N = 58 (25%)	
Sodium (mg)	6998 (5596, 9055)	7652 (6284, 9975)	0.079
Potassium (mg)	3396 (2732, 3839)	3291 (2546, 4081)	0.78
Calcium (mg)	1233 (1114, 1459)	1250 (1086, 1461)	0.72
Phosphorus (mg)	1447 (1270, 1656)	1486 (1294, 1688)	0.68
Magnesium (mg)	392 (334, 453)	373 (279, 433)	0.15
Iron (mg)	15.4 (13.8, 16.6)	15.7 (13.9, 17.4)	0.18
Zinc (mg)	9.50 (6.68, 11.75)	9.40 (7.22, 11.42)	0.66
Iodine (µg)	147 (81, 174)	141 (93, 167)	0.86
Copper (mg)	1.02 (0.76, 1.20)	1.03 (0.86, 1.19)	0.60
Chlorine (mg)	1793 (1507, 2412)	1822 (1501, 2525)	0.93
Manganese (mg)	2.73 (1.20, 3.66)	2.94 (1.56, 3.65)	0.054
Selenium (µg)	56 (47, 63)	57 (53, 67)	0.23

**Table 6:** Relationship between physical activity, stress level, and blood pressure groups.

Characteristic	NBP	HAG1	*p*-Value
	1 N = 172 (75%)	2 N = 58 (25%)	
Low physical activity level	150 (87%)	50 (86%)	0.84
Moderate physical activity level	22 (13%)	8 (14%)	
Stress score	10.00 (8.00, 13.00)	13.00 (10.00, 14.00)	<0.001

**Table 7:** Total Effect, Direct Effect, and Indirect Effect on mediation analysis for diastolic blood pressure in university students.

				95% C.I. (a)				
Type	Effect	Estimate	SE	Lower	Upper	β	z	*p*
Indirect	Stress 1 ⇒ Weight ⇒ PA Diastolic	0.365	0.2715	−0.1671	0.8972	0.02732	1.3445	0.179
	Stress 2 ⇒ Weight ⇒ PA Diastolic	1.113	0.3826	0.3631	1.8628	0.08022	2.9090	0.004
	Stress 3 ⇒ Weight ⇒ PA Diastolic	0.149	1.5880	−2.9636	3.2612	0.00152	0.0937	0.925
	Sex at birth 1 ⇒ Weight ⇒ PA Diastolic	−2.202	0.5918	−3.3615	−1.0419	−0.16900	−3.7205	<0.001
Component	Stress 1 ⇒ Weight	2.082	1.4583	−0.7758	4.9405	0.09021	1.4279	0.153
	Weight ⇒ PA Diastolic	0.175	0.0439	0.0892	0.2614	0.30285	3.9906	<0.001
	Stress 2 ⇒ Weight	6.348	1.4939	3.4205	9.2765	0.26488	4.2496	<0.001
	Stress 3 ⇒ Weight	0.849	9.0556	−16.8997	18.5978	0.00503	0.0938	0.925
	Sex at birth1 ⇒ Weight	−12.559	1.2205	−14.9510	−10.1666	−0.55802	−10.2896	<0.001
Direct	Stress 1 ⇒ PA Diastolic	−1.899	0.9759	−3.8119	0.0135	−0.14214	−1.9462	0.052
	Stress 2 ⇒ PA Diastolic	0.540	1.0337	−1.4858	2.5660	0.03893	0.5225	0.601
	Stress 3 ⇒ PA Diastolic	14.769	6.0335	2.9434	26.5941	0.15104	2.4478	0.014
	Sex at birth1 ⇒ PA Diastolic	0.653	0.9827	−1.2730	2.5790	0.05012	0.6645	0.506
Total	Stress 1 ⇒ PA Diastolic	−1.534	1.0068	−3.5076	0.4392	−0.11482	−1.5237	0.128
	Stress 2 ⇒ PA Diastolic	1.653	1.0314	−0.3685	3.6747	0.11915	1.6027	0.109
	Stress 3 ⇒ PA Diastolic	14.918	6.2523	2.6633	27.1719	0.15256	2.3859	0.017
	Sex at birth1 ⇒ PA Diastolic	−1.549	0.8427	−3.2004	0.1030	−0.11888	−1.8378	0.066

Note. Confidence intervals computed using the method: Standard (Delta method).

**Table 8:** Total Effect, Direct Effect, and Indirect Effect on mediation analysis for systolic blood pressure in university students.

				95% C.I. (a)				
Type	Effect	Estimate	SE	Lower	Upper	β	z	*p*
Indirect	Stress 1 ⇒ Weight ⇒ PA Systolic	0.587	0.4274	−0.250	1.425	0.03318	1.3740	0.169
	Stress 2 ⇒ Weight ⇒ PA Systolic	1.791	0.5508	0.711	2.870	0.09742	3.2507	0.001
	Stress 3 ⇒ Weight ⇒ PA Systolic	0.239	2.5545	−4.767	5.246	0.00185	0.0937	0.925
	Sex at birth1 ⇒ Weight ⇒ PA Systolic	−3.542	0.7817	−5.074	−2.010	−0.20523	−4.5311	<0.001
Component	Stress 1 ⇒ Weight	2.082	1.4583	−0.776	4.941	0.09021	1.4279	0.153
	Weight ⇒ PA Systolic	0.282	0.0559	0.173	0.392	0.36777	5.0468	<0.001
	Stress 2 ⇒ Weight	6.348	1.4939	3.420	9.276	0.26488	4.2496	<0.001
	Stress 3 ⇒ Weight	0.849	9.0556	−16.900	18.598	0.00503	0.0938	0.925
	Sex at birth 1 ⇒ Weight	−12.559	1.2205	−14.951	−10.167	−0.55802	−10.2896	<0.001
Direct	Stress 1 ⇒ PA Systolic	0.296	1.2414	−2.137	2.729	0.01674	0.2387	0.811
	Stress 2 ⇒ PA Systolic	3.123	1.3149	0.546	5.700	0.16992	2.3751	0.018
	Stress 3 ⇒ PA Systolic	16.128	7.6751	1.085	31.171	0.12450	2.1014	0.036
	Sex at birth 1 ⇒ PA Systolic	−0.474	1.2501	−2.924	1.976	−0.02748	−0.3794	0.704
Total	Stress 1 ⇒ PA Systolic	0.884	1.3054	−1.675	3.442	0.04992	0.6769	0.498
	Stress 2 ⇒ PA Systolic	4.914	1.3373	2.293	7.535	0.26733	3.6742	<0.001
	Stress 3 ⇒ PA Systolic	16.368	8.1064	0.479	32.256	0.12635	2.0191	0.043
	Sex at birth1 ⇒ PA Systolic	−4.016	1.0926	−6.158	−1.875	−0.23270	−3.6760	<0.001

Note. Confidence intervals computed using the method: Standard (Delta method).

## Data Availability

Access to the study information will be provided by the authors, though, the e-mails declared and sent to the researchers, in compliance with the Ethics Committee’s provision on the anonymization of information.

## References

[1] WHO Obesidad y Sobrepeso. https://www.who.int/es/news-room/fact-sheets/detail/obesity-and-overweight.

[2] World Obesity Federation (2023). World Obesity Atlas. https://data.worldobesity.org/publications/?cat=19.

[3] WOF The Economic Impact of Overweight & Obesity in 2020 and 2060. https://data.worldobesity.org/resources/WOF-Economic-Impacts-2-V2.pdf.

[4] Roth G.A., Mensah G.A., Fuster V. (2020). La carga global de las enfermedades y riesgos cardiovasculares: Una brújula para la acción global. J. Am. Coll. Cardiol..

[5] Miedlich S.U., Sahay P., Olivares T.E., Lamberti J.S., Morse D.S., Brazill K.P., Chhabra K.H., Bainbridge L. (2024). Lifestyle and mood correlates of cardiometabolic risk in people with serious mental illness on second-generation antipsychotic medications. PLoS ONE.

[6] Ganipisetti V.M., Bollimunta P. (2025). Obesity and Set-Point Theory. StatPearls [Internet].

[7] Dzau V.J., Hodgkinson C.P. (2024). Precision Hypertension. Hypertension.

[8] Chaturvedi A., Zhu A., Gadela N.V., Prabhakaran D., Jafar T.H. (2024). Social Determinants of Health and Disparities in Hypertension and Cardiovascular Diseases. Hypertension.

[9] Berdalin A.B., Namestnikova D.D., Cherkashova E.A., Golovin D.A., Gubskiy I.L., Lelyuk V.G. (2023). Arterial Hypertension and Its Consequences Are the Main Predictors of Embolic Stroke of Undetermined Source. Dis. Markers.

[10] Harbin M.M., Hultgren N.E., Kelly A.S., Dengel D.R., Evanoff N.G., Ryder J.R. (2018). Measurement of central aortic blood pressure in youth: Role of obesity and sex. Am. J. Hypertens..

[11] Ruilope L.M., Nunes Filho A.C.B., Nadruz W., Rodríguez Rosales F.F., Verdejo-Paris J. (2018). Obesity and hypertension in Latin America: Current perspectives. Hipertens. Riesgo Vasc..

[12] López de Fez C.M., Gaztelu M.T., Rubio T., Castaño A. (2004). Mecanismos de Hipertensión en Obesidad. An. Sist. Sanit. Navar..

[13] Khan W.B., Bhura S.A.B. (2024). The association between age of onset of obesity and the rising risk of hypertension a cause of concern for young adults. J. Pak. Med. Assoc..

[14] Blüher M. (2025). An overview of obesity-related complications: The epidemiological evidence linking body weight and other markers of obesity to adverse health outcomes. Diabetes Obes. Metab..

[15] García Casilimas G.A., Martin D.A., Martínez M.A., Merchán C.R., Mayorga C.A., Barragán A.F. (2017). Fisiopatología de la hipertensión arterial secundaria a obesidad. Arch. Cardiol. México.

[16] Bray G.A., Kim K.K., Wilding J.P.H., World Obesity Federation (2017). Obesidad: Un proceso crónico de enfermedad progresiva. Una declaración de posición de la Federación Mundial de Obesidad: Documento de posición. Obes. Rev..

[17] Yamada-Goto N., Sei N., Murai-Takeda A., Azegami T., Sakakibara-Adachi K., Hayashi K., Inokuchi M., Hirose H. (2025). Longitudinal impact of weight change on blood pressure in University students. Hypertens. Res..

[18] Saini V., Guada L., Yavagal D.R. (2021). Epidemiología global de la trazo y acceso a intervenciones agudas de accidente cerebrovascular isquémico. Neurología.

[19] World Health Organization (2012). Guideline: Sodium Intake for Adults and Children.

[20] Fang Z., Raza U., Song J., Lu J., Yao S., Liu X., Zhang W., Li S. (2025). Systemic aging fuels heart failure: Molecular mechanisms and therapeutic avenues. ESC Heart Fail..

[21] Gong H.J., Tang X., Zhou J.B. (2024). The association between weight change patterns and obesity-related complex multimorbidity: Evidence from NHANES. Front. Endocrinol..

[22] Guapi-Guamán F., Morcillo-Valencia R., Falcones-Barbosa E.D.R., Mina-Gonzáles J. (2022). Prevalencia de sobrepeso y obesidad. Problema de salud en la comunidad universitaria y politécnica ecuatoriana. Rev. Arbitr. Interdiscip. Cienc. Salud Salud Vida.

[23] Kobayashi T. (2003). Risk factors for ischemic heart disease in young adults. Nihon Rinsho.

[24] Kim J., Thayabaranathan T., Donnan G.A., Howard G., Howard V.J., Rothwell P.M., Feigin V., Norrving B., Owolabi M., Pandian J. (2020). Estadísticas de accidentes cerebrovasculares globales 2019. Rev. Int. Stroke.

[25] Wang Y., Ye C., Kong L., Zheng J., Xu M., Xu Y., Li M., Zhao Z., Lu J., Chen Y. (2023). Independent Associations of Education, Intelligence, and Cognition with Hypertension and the Mediating Effects of Cardiometabolic Risk Factors: A Mendelian Randomization Study. Hypertension.

[26] Adetunji A., Uche-Orji C., Ezebialu C., Adebayo P.C., Sanusi F., Imo U., Adiat T., Ajala T. (2025). The prevalence and risk factors of pre-hypertension and hypertension among clinical students at the university of Ibadan, Nigeria. BMC Cardiovasc. Disord..

[27] Mendoza-Herrera K., Pedroza-Tobías A., Hernández-Alcaraz C., Ávila-Burgos L., Aguilar-Salinas C.A., Barquera S. (2019). Attributable burden and expenditure of cardiovascular diseases and associated risk factors in Mexico and other selected mega-countries. Int. J. Environ. Res. Public Health.

[28] Garvey W.T., Mechanick J.I., Brett E.M., Garber A.J., Hurley D.L., Jastreboff A.M., Nadolsky K., Pessah-Pollack R., Plodkowski R. (2016). Asociación estadounidense de endocrinólogos clínicos y universidad americana de endocrinología guías de práctica clínica integral para la atención médica de pacientes con obesidad. Endocr. Pract..

[29] John J., Wisniewski P., Fieggen G., Kaestner L., Lazarus J. (2025). Intrarenal Pressure in Retrograde Intrarenal Surgery: A Narrative Review. Urology.

[30] Kovesdy C.P. (2025). Obesity and Metabolic Health in CKD. Clin. J. Am. Soc. Nephrol..

[31] Scott E.M., Carpenter J.S., Iorfino F., Cross S.P.M., Hermens D.F., Gehue J., Wilson C., White D., Naismith S.L., Guastella A.J. (2019). What is the prevalence, and what are the clinical correlates, of insulin resistance in young people presenting for mental health care? A cross-sectional study. BMJ Open.

[32] Morejón Terán Y.A., Manzano A.S., Betancourt Ortiz S., Ulloa V.A., Sandoval V., Espinoza Fajardo A.C., Carpio-Arias V. (2021). Construcción de un Cuestionario de Frecuencia de Consumo de Alimentos para Adultos Ecuatorianos, estudio transversal. Rev. Esp. Nutr. Humana Diet..

[33] Software de Nutrición. https://www.nutrimind.net/.

[34] Bauce G., Moya-Sifontes M.Z. (2020). Waist circumference weight index as a complementary indicator of overweight and obesity in different groups of subjects. Rev. Dig. Postgrad..

[35] Gómez-León Mandujano A., Morales López S., Álvarez Díaz C.D.J. (2016). Técnica para una correcta toma de la presión arterial en el paciente ambulatorio. Rev. Fac. Med..

[36] Garmendia M., Lera L., Sánchez H., Albala C. (2009). Valores normativos de resistencia a la insulina mediante HOMA-IR en adultos mayores de Santiago de Chile. Rev. Méd. Chile.

[37] Román F., Santibáñez P., Vinet E.V. (2016). Uso de las Escalas de Depresión Ansiedad Estrés (DASS-21) como Instrumento de Tamizaje en Jóvenes con Problemas Clínicos. Acta Investig. Psicol..

[38] IPAQ-TM.pdf. https://youthrex.com/wp-content/uploads/2019/10/IPAQ-TM.pdf.

[39] Jamovi. https://www.jamovi.org/about.html.

[40] Talati K.N., Parmar A., Zalavadiya D., Shinde M., Madan-Patel G. (2022). Epidemiological Insights into Anthropometric Indices and Their Correlates among College Students through a University-Level Screening Program in Western India. Indian J. Community Med..

[41] Rangel Caballero L.G., Murillo López A.L., Pulido Silva G. (2021). Asociación entre el sobrepeso y la obesidad con el colesterol, la presión arterial y la diabetes en estudiantes universitarios panameños. Rev. Cuba Investig. Bioméd.

[42] Hemmingsson E., Ekelund U. (2007). Is the association between physical activity and body mass index obesity dependent?. Int. J. Obes..

[43] Yarizadeh H., Setayesh L., Roberts C., Yekaninejad M.S., Mirzaei K. (2022). Nutrient pattern of unsaturated fatty acids and vitamin E increase resting metabolic rate of overweight and obese women. Int. J. Vitam. Nutr. Res..

[44] Yarizadeh H., Setayesh L., Majidi N., Rasaei N., Mehranfar S., Ebrahimi R., Casazzza K., Mirzaei K. (2022). Nutrient patterns and their relation to obesity and metabolic syndrome in Iranian overweight and obese adult women. Eat. Weight Disord..

[45] Torres K., Cáceres-Durán M.A., Orellana C., Osorio M., Simón L. (2025). Nutritional imbalances among university students and the urgent need for educational and nutritional interventions. Front. Nutr..

[46] Koncar D., Kovacevic A., Miler M., La Grasta Sabolic L., Dika Z., Softic D., Valent Moric B. (2024). Understanding the Impact of Obesity and Parental Blood Pressure in Identifying Optimal Hypertension Screening Group in Youth. Cureus.

[47] Fang Y., Tavengana G., Wu H., Mei W., Jiang C., Wang C., Ren X., Hu J., Su F., Cheng S. (2024). Elevated blood pressure and hyperuricemia risk: A retrospective cohort study from Wuhu, China. Sci. Rep..

[48] Sadabadi F., Talkhi N., Omouri-Kharashtomi M., Mirzaei M., Saffar Soflaei S., Rahimi Z., Shabani N., Latifi M., Mohammadtaghizadeh Sarabi M., Iri S. (2025). Association Between Socio-Economic Status (SES) and the Traditional Risk Factors of Cardiovascular Diseases (CVD): A Cross-Sectional MASHAD Cohort Study Results. Health Sci. Rep..

[49] Bryce-Moncloa A., Alegría-Valdivia E., San Martin-San Martin M.G. (2017). Obesidad y riesgo de enfermedad cardiovascular. An. Fac. Med..

[50] Niu Z.J., Cui Y., Wei T., Dou M., Zheng B.X., Deng G., Tian P.X., Wang Y. (2024). The effect of insulin resistance in the association between obesity and hypertension incidence among Chinese middle-aged and older adults: Data from China health and retirement longitudinal study (CHARLS). Front. Public Health.

[51] Li M., Ji R., Liu X., Wu Y. (2024). Associations of metabolic syndrome and its components with sarcopenia, and the mediating role of insulin resistance: Findings from NHANES database. BMC Endocr. Disord..

[52] Wankhar D., Prabu Kumar A., Vijayakumar V., Velan A., Balakrishnan A., Ravi P., Rudra B., Maheshkumar K. (2024). Effect of Meditation, Mindfulness-Based Stress Reduction, and Relaxation Techniques as Mind-Body Medicine Practices to Reduce Blood Pressure in Cardiac Patients: A Systematic Review and Meta-Analysis. Cureus.

[53] Keogh T.M., Howard S. (2024). Social participation is associated with a habituated blood pressure response to recurrent stress. Int. J. Psychophysiol..

[54] Alolabi H., Alchallah M.O., Mohsen F., Marrawi M., Alourfi Z. (2022). Social and psychosocial factors affecting eating habits among students studying at the Syrian Private University: A questionnaire based cross-sectional study. Heliyon.

[55] Tyra A.T., Young D.A., Ginty A.T. (2023). Emotion regulation tendencies and cardiovascular responses to repeated acute psychological stress. Int. J. Psychophysiol..

[56] Manne-Goehler J., Fabian J., Sokhela S., Akpomiemie G., Rahim N., Lalla-Edward S.T., Brennan A.T., Siedner M.J., Hill A., Venter W.D.F. (2024). Blood pressure increases are associated with weight gain and not antiretroviral regimen or kidney function: A secondary analysis from the ADVANCE trial in South Africa. J. Int. AIDS Soc..

[57] Hoek A.G., van Oort S., Elders P.J.M., Beulens J.W.J. (2022). Causal Association of Cardiovascular Risk Factors and Lifestyle Behaviors with Peripheral Artery Disease: A Mendelian Randomization Approach. J. Am. Heart Assoc..

[58] Sunday O.G., Okorie S.L., Ogugua E.A., Muracki J., Kurtoglu A., Alotaibi M.H., Elkholi S.M. (2024). Relationship of anthropometrics and blood pressure to identify people at risk of hypertension and obesity-related conditions in Nigerian rural areas. Medicine.

[59] Hu W., Liu B.P., Jia C.X. (2024). Association and biological pathways between lung function and incident depression: A prospective cohort study of 280,032 participants. BMC Med..

[60] Feng J., Wu Y., Meng M., Zeng R., Ma Y., Luo D., Zhang L., Zhang Y., Li Y., Huang W. (2024). The mediating effect of blood biomarkers in the associations between inflammatory bowel disease and incident psychiatric disorders: A prospective cohort study. Int. J. Surg..

[61] Cui C., Liu L., Li H., Qi Y., Song J., Han N., Wang Z., Shang X., Sheng C., Balmer L. (2024). Childhood Exposure to Interparental Physical Violence and Adult Cardiovascular Disease. JAMA Netw. Open.

[62] Yu S., Guo X., Li G., Yang H., Zheng L., Sun Y. (2022). Low educational status correlates with a high incidence of mortality among hypertensive subjects from Northeast Rural China. Front. Public Health.

